# Epstein-Barr Virus Infection of Polarized Epithelial Cells via the Basolateral Surface by Memory B Cell-Mediated Transfer Infection

**DOI:** 10.1371/journal.ppat.1001338

**Published:** 2011-05-05

**Authors:** Claire Shannon-Lowe, Martin Rowe

**Affiliations:** Cancer Research UK Birmingham Cancer Centre, School of Cancer Sciences, College of Medical and Dental Sciences, The University of Birmingham, Birmingham, United Kingdom; University of Wisconsin-Madison, United States of America

## Abstract

Epstein Barr virus (EBV) exhibits a distinct tropism for both B cells and epithelial cells. The virus persists as a latent infection of memory B cells in healthy individuals, but a role for infection of normal epithelial is also likely. Infection of B cells is initiated by the interaction of the major EBV glycoprotein gp350 with CD21 on the B cell surface. Fusion is triggered by the interaction of the EBV glycoprotein, gp42 with HLA class II, and is thereafter mediated by the core fusion complex, gH/gL/gp42. In contrast, direct infection of CD21-negative epithelial cells is inefficient, but efficient infection can be achieved by a process called transfer infection. In this study, we characterise the molecular interactions involved in the three stages of transfer infection of epithelial cells: (i) CD21-mediated co-capping of EBV and integrins on B cells, and activation of the adhesion molecules, (ii) conjugate formation between EBV-loaded B cells and epithelial cells via the capped adhesion molecules, and (iii) interaction of EBV glycoproteins with epithelial cells, with subsequent fusion and uptake of virions. Infection of epithelial cells required the EBV gH and gL glycoproteins, but not gp42. Using an *in vitro* model of normal polarized epithelia, we demonstrated that polarization of the EBV receptor(s) and adhesion molecules restricted transfer infection to the basolateral surface. Furthermore, the adhesions between EBV-loaded B cells and the basolateral surface of epithelial cells included CD11b on the B cell interacting with heparan sulphate moieties of CD44v3 and LEEP-CAM on epithelial cells. Consequently, transfer infection was efficiently mediated via CD11b-positive memory B cells but not by CD11b–negative naïve B cells. Together, these findings have important implications for understanding the mechanisms of EBV infection of normal and pre-malignant epithelial cells *in vivo*.

## Author Summary

Epstein-Barr virus (EBV) is an important human pathogen that is carried as a latent infection of B cells by most adults worldwide. Infection of epithelial cells is also believed to be important in the normal life-cycle of EBV, and is certainly associated with the pathogenesis of some epithelial tumours. Whilst EBV binds to and infects B cells that express CD21, a receptor for the gp350 viral glycoprotein, binding of EBV to CD21-negative epithelial cells is inefficient. However, we have identified an efficient process of ‘transfer infection’. This process involves EBV first binding to B cells, resulting in CD21-mediated capping of virus and activation of adhesion molecules, which facilitates conjugate formation between B cells and epithelial cells and the subsequent entry of EBV into epithelial cells. We have characterised the molecular processes involved in transfer infection, both in unpolarized cells modelling pre-malignant epithelial cells, and in normal polarized epithelial cells. The details of the molecular interactions in these infection models led us to identify a subset of B cells, memory B cells, as being the primary vehicles for transfer infection. The results reinforce the likely physiological significance of transfer infection of epithelial cells in healthy persistence and in EBV pathogenesis.

## Introduction

Epstein Barr virus (EBV) is a ubiquitous human herpesvirus that exhibits a distinct tropism for both B cells and epithelial cells. Thus, EBV establishes lifelong latent infection in the memory B cell pool, and has a strong association with B cell tumours, including post-transplant lymphoproliferative disease, Burkitt's lymphoma and Hodgkin's lymphoma. EBV is also present in epithelial cells of oral hairy leukoplakia, undifferentiated nasopharyngeal carcinoma, and some gastric carcinomas, and infection of epithelial cells in asymptomatic persistent EBV infection is thought to be a feature of the normal EBV life-cycle [1]. Whilst the process of B cell infection by EBV is well characterised, the natural mechanisms of epithelial cell infection are poorly understood.

Infection of B cells by EBV is very efficient. It is initiated by the interaction of the major viral envelope glycoprotein, gp350, with the complement receptor, CD21 (also known as CR2), on the B cell surface [2], [3]. Fusion and internalisation of the virus is triggered by the interaction of a second envelope glycoprotein, gp42, with HLA class II [4], and is thereafter mediated by the core fusion complex, gH/gL/gp42 [5], [6].

In contrast to B cells, epithelial cells were thought to lack expression of CD21 molecules; although CD21 mRNA transcripts have been detected in some tonsillar epithelial cells [7], the CD21 protein is not normally detected. Consequently, infection of epithelial cells with cell-free virus *in vitro* is highly variable [8] and may necessitate the use of supraphysiological amounts of virus [9]. Infection can be improved *in vitro* by co-culturing the epithelial cells with the Akata B cell line induced into lytic replication [10]. In addition, we recently described a process of transfer infection whereby EBV can efficiently access the epithelium by first binding to resting B cells which act as a transfer vehicle to infect epithelial cells [11]. Transfer infection involves three stages: (i) CD21-mediated capping of EBV on the B cell surface, (ii) conjugate formation between EBV-loaded B cells and epithelial cells, and (iii) virus fusion and uptake by epithelial cells. The molecular interactions involved in the process of conjugate formation and transfer of EBV into the epithelial cells remain undefined. Nevertheless, the efficient infection of epithelial cells lacking expression of HLA class II and CD21 indicates a fundamental difference between viral entry in B cells and in epithelial cells.

The present work addresses two issues; what might be the physiological relevance of transfer infection, and what are the molecular mechanisms involved?

With regards to the physiological relevance of transfer infection in the normal life-cycle of EBV infection, we first need to consider when epithelial cells might become infected. EBV is normally transmitted to a new host via salivary secretions to the oropharynx. If the virus is unable to infect epithelial cells directly, then it must somehow traverse the epithelial membrane barrier of the host to access B cells, perhaps via physical wounds to the epithelium or as a result of inflammation-induced leakiness. Having bound to a B cell, the incoming virus has the opportunity to infect and colonise the B cell compartment. It is not known to what extent or whether epithelial cells become infected when the host first encounters EBV during primary infection. However, once latency is established in the memory B cell compartment there must be mechanisms for activation of lytic cycle to maintain the pool of virus infected B cells and to produce the virus that is regularly detected in salivary secretions. It has long been argued, primarily by extrapolation of the observations with oral hairy leukoplakia in AIDS patients where the lesions on the tongue represent foci of epithelial cells supporting lytic EBV replication, that virus released from rare reactivated EBV-carrying B cells somehow infects epithelial cells which differentiate and initiate lytic cycle. In this model, epithelial cell infection is proposed to be an amplification step and a mechanism for EBV to traverse the epithelial barrier to reach the oral secretions.

We hypothesise that transfer infection offers a mechanism for how EBV can infect epithelial cells during persistent infection. It does, however, predicate that EBV-loaded B cells come into contact and form conjugates with epithelial cells in the oral tissues. The immune system is dependent upon the rapid mobilization of leucocytes to sites of inflammation. This mobilization necessitates the movement of vascular leukocytes through endothelial barriers in an apical to basolateral direction, and occurs via a co-ordinated, multistep sequence of adhesion events. Adhesion to endothelial cells involves tethering, rolling, firm adhesion and extravasation, and is mediated by L-selectin, LFA-1 and VLA-4 on B cells binding to their cognate receptors PNAD, ICAM-1 and VCAM-1 respectively on the apical surface of endothelial cells [12], [13]. In contrast, the first normal epithelial surface that B cells will encounter following extravasation is the basolateral surface. Lytic virus production in B cells has been observed in secondary lymphoid tissues such as tonsils, usually in the extra-nodal sub-epithelial areas [14]. It might be expected, therefore, that transfer infection of epithelial cells via B cells is likely to occur at the basolateral surface of the epithelium. However, in contrast to the well-studied interactions of B-lymphocytes with endothelial cells, interactions of B cells with epithelial cells are poorly understood.

The case for the physiological relevance of transfer infection would be strengthened if the molecular mechanisms of transfer infection were understood and could be related to the anatomy of the lympho-epithelial tissues of the oropharynx. In the present report, therefore, we sought to determine which molecules were involved in capping of EBV on the B cell surface, what were the essential adhesion interactions involved in B cell/epithelial cell conjugate formation, and which viral proteins were required for infection of epithelial cells.

These studies were performed both with conventional monolayer cultures of unpolarized epithelial cells, which may model the scenario of infection of pre-malignant cells, and with polarized primary undifferentiated oropharyngeal epithelial cells which may more closely model infection of normal epithelia during persistent infection of the healthy host. We identified adhesion molecule interactions involved in conjugate formation, which differed between unpolarized and polarized epithelial cells. We also demonstrated that transfer infection of polarized cells could only be achieved via the basolateral surface. Not only were cellular adhesion molecules differentially located at the apical and basolateral surfaces of polarized cells, but so were the virus receptors necessary for EBV entry into epithelial cells. Finally, the experiments identified the CD11b+ memory B cell subset as the prime vehicle for transfer infection through binding to the heparan sulphate moieties of CD44v3 and lymphocyte-endothelial-epithelial cell adhesion molecule (LEEP-CAM) at the basolateral surface.

## Materials and Methods

### Cells and cell lines

Primary B cells were isolated from peripheral blood using CD19 Dynabeads and CD19 detachabeads (Invitrogen), as described previously [15]. Cultures of primary tonsillar epithelial cells were generated as described previously [16]. The cells were resuspended in 10 ml serum-free keratinocyte growth medium (Invitrogen Gibco) containing 1% penicillin/streptomycin (Sigma), 0.2% fungizone (Sigma). Ethical approval for the use of primary B and epithelial cells was granted by The South Birmingham Research Ethics committee, 07/H1207/246. All tonsillar epithelial cells were used at passage 1 or 2.

### Generation of a polarized epithelial cell monolayer

Primary tonsillar epithelial cells at passage 1 were seeded at 2×10^4^ cells on the upper surface or 4×10^4^ cells on the lower surface of Transwell permeable PET membranes (8.0 µm pore size; Costar). To seed the cells on the lower surface, the transwell was inverted for two hours to allow the cells to attach. The cells reached confluence at 2 to 3 days post seeding and the monolayers were grown for at least a further 6 days with fresh medium added every 2 days. Permeability of the polarized monolayer was quantified by measuring the transepithelial flux of a 4-kDa fluorescein isothiocyanate (FITC)-labelled dextran molecule (Sigma Aldrich). Briefly, following polarization, 200 µg of FITC-dextran was applied to the apical surface of the monolayer. Following incubation at 37°C for 3 hours, a 200 µl aliquot of the medium from the basolateral compartment was analysed for FITC fluorescence (BioRad plate Reader).

### Virus preparations and quantitation of EBV genome load

Preparations of the 2089 recombinant wild type EBV with a GFP insert [17] or viruses deleted for gp350 [18], gp42 and gp85 [11], (kindly provided by Professor Delecluse, German Cancer Research Centre, Heidelberg) were made from 293 cells carrying the recombinant B95.8 EBV genomes, and transfected with a BALF4 expression plasmid to optimize gp110 levels [19]; gp110 low virus refers to virus not optimised with the BALF4 expression plasmid. Complementation of recombinant viruses deleted for the glycoproteins gp350, gp42 and gp85 was achieved by co-transfection of expression vectors encoding the respective deleted glycoproteins. Encapsidated and enveloped virus was purified from culture supernatants by centrifugation on an Optiprep (Axis Shield) self generated gradient [16] and quantitated by a Q-PCR assay amplifying a single copy gene, BALF5, using Namalwa cell line (2 integrated copies of EBV) as the standard [15]. The MOI of virus infections was defined as the number of EBV genomes in the purified virus preparations divided by the number of target cells in culture.

### Conjugate formation

Freshly isolated primary B cells were either uninfected, exposed to EBV for 24 hours or exposed to an agonistic anti-CD21 mAb reported to bind to the C3d binding domain of CD21 (BL13, Immunotech) for 24 hours prior to labeling with the tracking dye, PKH26 (Sigma Aldrich), according to the manufacturer's instructions. Epithelial cells were labelled with 10 nM CFSE (carboxyfluorescein diacetate succinimidyl ester; Molecular Probes) for 10 minutes at 37°C. Conjugate formation between the two cell types was performed as described [11].

### Transfer infection

Freshly isolated primary B cells were incubated with purified virus supernatant at an MOI of 100 for 18 to 24 hours at 37°C. The virus-loaded B cells were then applied to epithelial cultures for 1 hour at 37°C, then washed off, as previously described [11]. In experiments where virus-loaded B cells were applied to the basolateral surface of polarized cells via transwell filters, accurate quantitation of epithelial cell infection was determined by trypsinisation of the infected monolayers from the filters, removal of the B cells from the cell suspension with CD19-Dynabeads, and re-plating the epithelial cells into conventional culture wells of 24-well plates. All assays were performed on epithelial cell preparations from up to 4 donors unless otherwise stated.

### Inhibition of transfer infection

Freshly isolated primary B cells were incubated with purified virus as above. The virus-loaded B cells and/or epithelial cells were pre-incubated in up to 20 µg/ml (determined by antibody titration and previously published concentrations) of blocking antibody for 30 minutes at 4°C. The blocking antibodies include ICAM-1 (mAb 15.2, CR-UK), LFA-1 (mAb 38, CR-UK), beta-1 integrin (P5D2, R&D Systems, 6S6, Chemicon), CD11b (ICRF44, Biolegend), αVβ5 (p1f6, Chemicon), α5β1 (jbs5, Millipore), anti LEEP-CAM (6F10, kindly provided by M.Brenner, Department of Medicine, Harvard Medical School, Boston). To examine the involvement of integrins in transfer infection by blocking antibodies to integrins, the B cell and epithelial cells were initially washed in Ca^2+^/Mg^2+^-free PBS, and the co-cultures were performed in basic binding buffer (PBS, 0.5% FCS, 1g/L D-glucose, 2 mM Mg^2+^, 0.5 mM Ca^2+^), with the appropriate mAbs. To examine cation usage, the co-cultures were performed in binding buffer with the cations added individually or together at the above concentrations. Additionally, EDTA or EGTA were added at 5 mM or 2 mM respectively with appropriate cations. Heparan sulphate, chondroitin sulphate and hyaluronic acid (0–128 µg/ml, Sigma) were titrated out in binding buffer. Similarly, RGD peptides and scrambled peptides (0–200 µg/ml, Sigma) were titrated. B cells and epithelial cells were initially washed in Ca^2+^/Mg^2+^-free PBS, then pre-incubated in each dilution for 30 minutes at 37°C before co-culture with the epithelial cells. The extracellular matrix components Fibronectin (25 µg/ml) and Vitronectin (25 µg/ml, Sigma), rhICAM-1/Fc Chimera (25 µg/ml, R&D Systems) and Laminin β-1 (25 µg/ml, Millipore) were diluted in binding buffer and pre-incubated with the purified Δgp350 virus.

### Immunofluorescence, immunohistochemistry and confocal microscopy

Primary tonsillar epithelial cells were either grown to confluence on glass coverslips or polarized on Transwell PET membranes, and fixed in 2% paraformaldehyde for 30 minutes at room temperature or in 100% ice-cold methanol. Cells were permeabilised for 5 minutes in 0.5% Triton-X-100 and incubated with the primary antibodies to CD81 (M83), CD82 (TS82) and CD63 (6H1) kindly provided by Dr Berditchevski (The University of Birmingham), ICAM-1 (abCAM), CD44v3 (R&D systems), ZO-1 (Zymed), E-cadherin (abCAM), anti-beta-1 integrin (PB1 or P5D2; R&D Systems), anti-LFA-1 (abCAM), anti-gp350 (72A1), anti-CD21 (Pharmingen), αVβ6 (10d5, Chemicon), LFA-1 24 (abCAM) for 1 hour at room temperature. The cells were then washed and incubated with the relevant goat anti-mouse, rabbit or rat secondary antibody conjugated with Alexafluor 488, 555 or 647 (Invitrogen) at 1:1000 dilution. When unconjugated antibodies derived from the same species were used in co-staining experiments, the individual antibodies were pre-labeled with different fluorochromes using the Zenon antibody labeling kit (Invitrogen), as previously described [11]. The cells were mounted in Vectorshield-DAPI (Vector Laboratories) and examined by confocal microscopy.

### Electron microscopy

Primary epithelial cells were grown on 25 mm round gridded, numbered cover slips (Electron Microscopy Sciences). Virus-loaded and PKH26 dye-labeled B cells were co-cultured with the epithelial cells for 1 hour then the unbound cells gently washed off. The B cell-epithelial cell conjugates were initially examined by light and epi-fluorescence microscopy for pre-labelled B cells conjugated to unlabelled and larger epithelial cells, to determine the numbered location of the conjugates within the grid. The cells were then fixed in 2% paraformaldehyde/1.5% glutaraldehyde in 0.1 M sodium phosphate buffer, pH 7.4 for 20 minutes, post-fixed in 1.5% potassium ferricyanide, 1% osmium for 1 hour on ice and treated in 1% tannic acid for 45 minutes at room temperature. The cells were then dehydrated in graded ethanol (70%, 90% and absolute) then embedded in Epon 812 (TAAB laboratories). Ultrathin sections were stained with uranyl acetate [20].

## Results

### EBV induces firm adhesion between B cells and epithelial cells via activation of CD21

**Figure 1:**
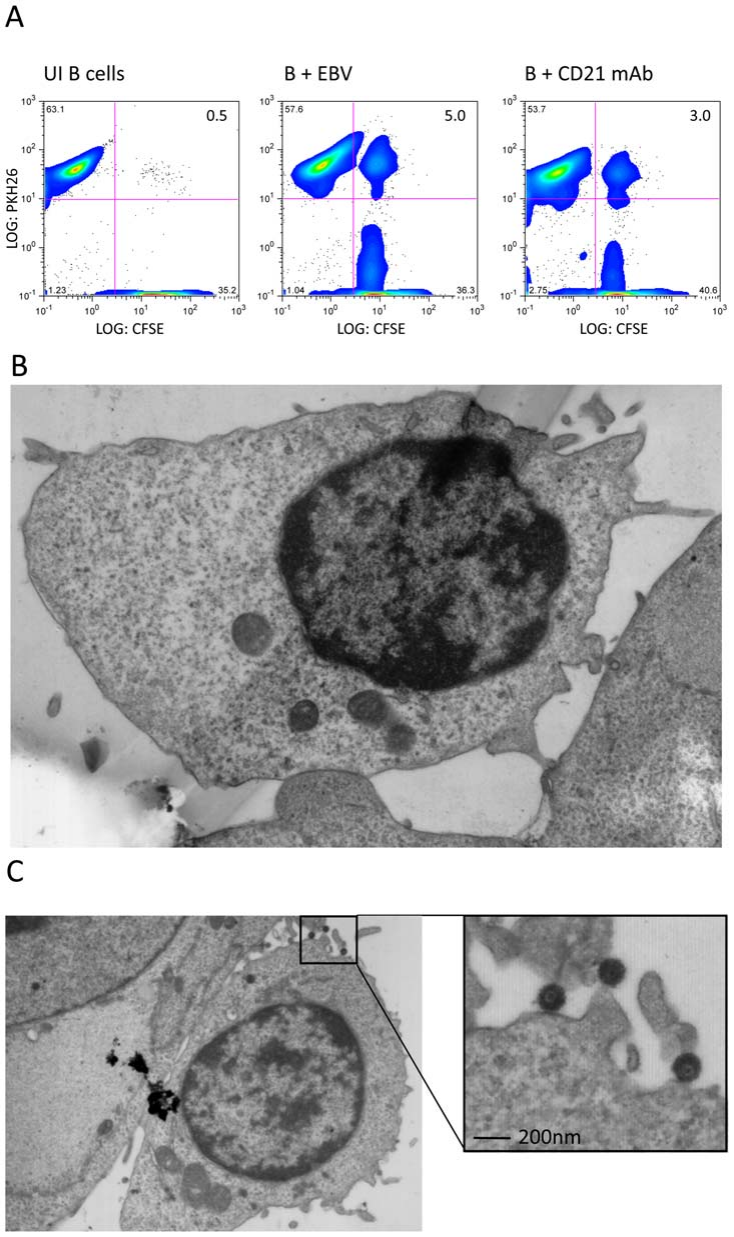
EBV induces firm adhesion between B cells and epithelial cells. (A) FACS profiles of conjugate formation between CFSE-labelled primary tonsillar epithelial cells (x-axis) and PKH26-labelled primary B cells (y-axis). The B cells were uninfected, EBV-infected (MOI 100, 24 h p.i.) or incubated with agonist mAb to CD21 (BL13). Conjugates appear in the upper right quadrant and the percentages of cells are shown. (B-C) Electron micrographs of B cell-epithelial cell conjugates. EBV-infected B cells (24 h p.i.) were co-cultured with primary tonsillar epithelial cells for 1 hour and immediately fixed, embedded in Epon 812, stained with uranyl acetate and ultra-thin sectioned. (B) The sites of interaction between the cell types show firm interaction. (C) A second conjugate revealing a firm interaction between the B- and epithelial cell and the fine ultrastructure of the virus particles.

Both EBV and complement C3d bind to the complement receptor CD21 (CR2) expressed on the B cell surface. Similarly to the effect of C3d, EBV binding to CD21 induces lymphocyte activation and capping of virus and CD21 with other B cell surface molecules such as tetraspanins and adhesion molecules. EBV-activated B cells can then form conjugates with epithelial cells via molecules in the cap, as we have previously reported [11]. Since EBV and complement bind to a similar region of the CD21 molecule, we asked whether B cell/epithelial cell conjugates might also be induced by an agonistic CD21 monoclonal antibody that also induces capping and CD21-mediated cell signaling [21]. The results of a representative conjugate assay shown in Figure 1A clearly demonstrated that capping of CD21 in the absence of EBV was sufficient to induce conjugate formation. Whilst uninfected B cells did not form conjugates with epithelial cells in this assay, 3 to 5% of EBV-infected or anti-CD21-stimulated B cells formed conjugates with epithelial cells. Furthermore, confocal microscopy showed that as with EBV-induced B cell/epithelial cell conjugates, the point of contact between the two cell types corresponded to the site of capped CD21 molecules (data not shown). Together, these results suggest that EBV hijacks the complement pathway in order to cap and activate the B cell adhesion molecules to enable interaction with the epithelial cells.

To examine the physical nature of the B cell/epithelial cell interaction during EBV transfer infection, we examined the ultrastructure of the virological synapse by electron microscopy. Epithelial cells were seeded onto coverslips with numbered grids and were co-cultured with virus-loaded B cells for 1 hour. Following removal of excess unbound B cells by washing, the remaining cells were fixed and embedded in preparation for examining ultrathin sections by electron microscopy. The sites of interaction between B cells and epithelial cells were observed to be in very close apposition, as illustrated in Figures 1B and 1C. Figure 1C shows an electron micrograph of a B cell/epithelial conjugate revealing a firm interaction between the two cells together with virus particles between the B cell-epithelial cell conjugate. These results strongly suggest that the interaction between each cell is both specific and mediated by activated adhesion molecules, resulting in a firm adhesion between the cells and thereby enabling transfer of the virus to the epithelial cell.

### B cell surface molecules present in the virological synapse

**Figure 2 ppat-1001338-g002:**
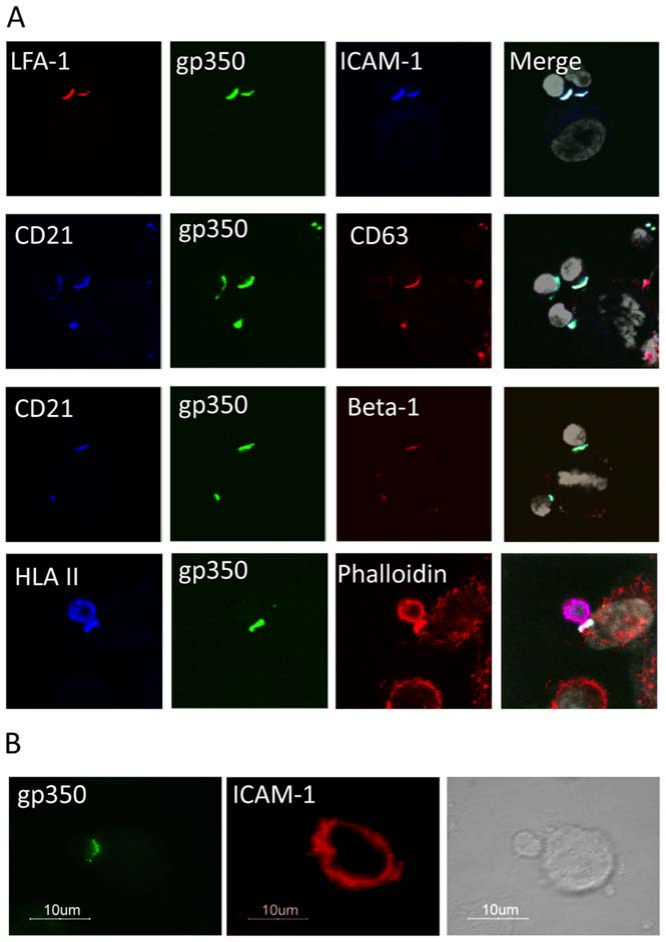
Analysis of B cell surface molecules at the virological synapse. (A) Confocal images of 1 µm Z slices through conjugates formed between EBV-infected B cells (24 hr p.i.) and primary tonsillar epithelial cells. Before co-culture, B cells were pre-labeled with non-blocking antibodies to virus (gp350) or cell surface molecules labeled with AlexaFluor 488 (green), 555 (red), 647 (blue) and DAPI (white). Antibodies derived from the same species were pre-labelled using the Zenon antibody-labeling kit. All gp350 staining co-localises at the cell-cell junction with the B cell surface molecules CD21, LFA-1, ICAM-1, CD63 and β-1 integrin. B cell surface HLA class II molecules are not restricted to the cell-cell junction, but are also present around the B cell surface. (B) Conjugates labelled with gp350 and ICAM-1 following co-culture showed the epithelial cell surface molecules did not relocate to the site of B cell-epithelial cell interaction.

To examine which B cell surface molecules were present only within the B cell-epithelial cell synapse, virus-loaded B cells were pre-labelled with a panel of non-blocking mAbs to cell surface molecules. These pre-labelled B cells were co-cultured with conventional non-labelled epithelial cell monolayer cultures for 1 hour before visualization by confocal microscopy. As illustrated in Figure 2A and summarized in Table 1, members of the CD21 complex (CD21, CD81, CD19) all co-capped with EBV (gp350) and were observed to be restricted to the synapse between B cell/epithelial cell conjugates. Likewise, tetraspanins (CD82, CD63), integrins (LFA-1, integrin β1, CD11b, integrin αvβ6) and Ig superfamily (ICAM-1, CD48) members were also found to co-cap with the EBV/CD21 complex. A selection of control molecules (e.g. HLA class I and class II, CD20, CD79a, CD79b, IgM and IgG) remained evenly distributed around the B cell surface and were not restricted to the area making contact with epithelial cells (Table 1). In contrast to the co-capping of various molecules on the B cell surface, the molecules on the epithelial cell surface did not co-cap at the B cell-epithelial cell junction. This is illustrated for ICAM-1 in Figure 2B where all cells were labeled after conjugate formation.

**Table 1:** B cell surface molecules capped following EBV infection and present within the B cell-epithelial cell synapse.

Restricted to Synapse						
gp350	gp42					Virus
CD21	CD19	CD81				Virus receptor
CD82	CD63					Tetraspanins
LFA-1	β1	CD11b	CD49d	αVβ6	CD47	Integrins
ICAM-1	CD48					Ig Superfamily

Not Restricted to Synapse
HLA class I and II
CD20
IgM, IgG
CD79a, CD79b

Notably, incubation of uninfected primary resting B cells with the agonistic anti-CD21 mAb (BL13), which binds to the complement-binding site on CD21, caused co-capping of exactly the same set of B cell molecules that were affected by binding of EBV (data not shown). Furthermore, not all CD21 was internalized following EBV binding or anti-CD21 mAb binding, as seen by the presence of cell-surface CD21 days after EBV-infection or mAb binding, probably because of lack of B cell receptor engagement [22].

Together, these data strongly suggest that EBV uses the same signaling pathways induced by complement binding to activate integrins and induce the B cells to interact with the epithelial cells.

### Adhesion molecules mediating B cell adhesion to epithelial cells during transfer infection

Integrins are a large family of cell surface αβ heterodimeric adhesion receptors that mediate intercellular adhesion, and adhesion of cells to components of the extracellular matrix. Integrin function is modulated by divalent cations including Ca^2+^, Mn^2+^ and Mg^2+^, and the interaction of certain integrins with their cognate receptors requires an Arg-Gly-Asp (RGD)-binding motif on the receptor. Given these parameters, by regulating cation availability and by blocking RGD-interactions, we investigated whether the firm adhesion observed between B cells and epithelial cells was mediated by integrins.

**Figure 3:**
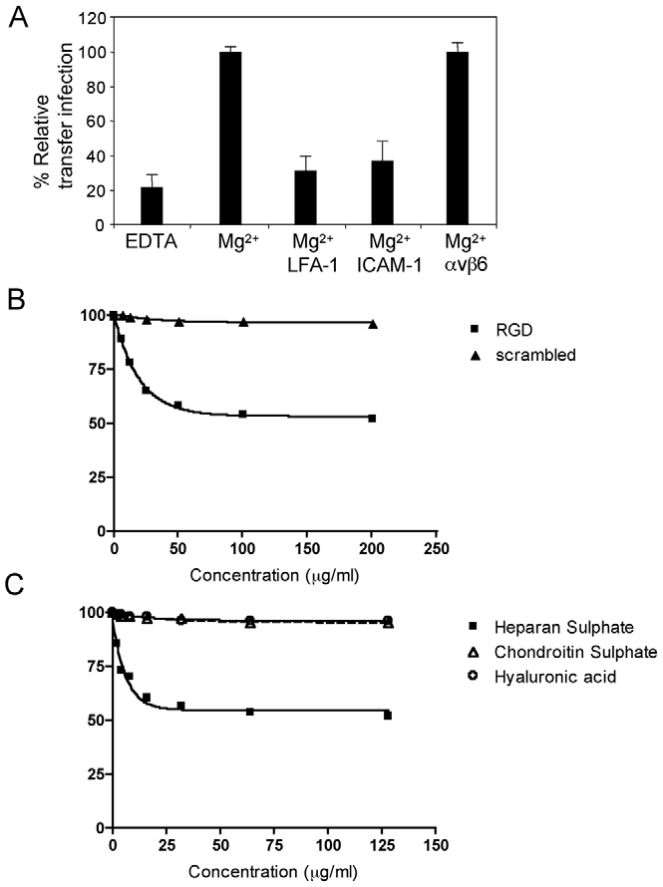
Molecules mediating B cell adhesion to epithelial cells. B cell-epithelial cell adhesion was examined by transfer infection for cation, RGD or GAG usage. (A) EBV-infected B cells (24 h p.i.) and tonsillar epithelial cells were washed in Ca^2+^/Mg^2+^-free PBS. Co-culture was performed in binding buffer in the absence of cations (EDTA) or the presence of Mg^2+^, then under optimal cation conditions, B cells and epithelial cells were pre-incubated in blocking antibodies to LFA-1, ICAM-1 and a control αvβ6. B cells were washed off after 1 hour of co-culture and epithelial cell infection (GFP) was examined by flow cytometry after 24 hours. The % GFP +ve epithelial cells were plotted as the mean of triplicates relative to the optimal transfer infection conditions. (B) RGD peptide and a scrambled control peptide were titrated and pre-incubated with the B cells and epithelial cells under optimal cation conditions in binding buffer. Co-culture was performed for 1 hour and epithelial cell infection analysed as above. The mean % GFP +ve of triplicate epithelial cell infections were plotted against the peptide concentration. (C) The glycosaminoglycans (GAGs) heparan sulphate, hyaluronic acid and chondroitin sulphate were titrated as above. The mean % GFP +ve of triplicate epithelial cells infections were plotted against the GAG concentrations.

We initially set up transfer infection experiments with conventional epithelial cell monolayer cultures and virus-loaded B cells. Both donor and target cells were washed twice in Ca^2+^/Mg^2+^-free PBS, then co-cultured with EDTA to scavenge residual cations, or with Mg^2+^ to restore integrin function. The cells were co-cultured for 1 hour in the same conditions before washing away donor B cells, and infection of epithelial cells was quantitated after 24 hours by flow cytometric analysis of GFP expression. Given that primary B cells do not express GFP following EBV infection until at least day 4, only GFP-positive epithelial cells were quantitated. Furthermore, epithelial cells and B cells were readily distinguished by gating on Forward/Side scatter parameters (supplementary Figure S1). The efficiency of transfer infection was shown to be substantially reduced by 80% relative to control levels in the absence of divalent cations (Figure 3A). Furthermore, pre-incubation of the cells with blocking antibodies to LFA-1 or ICAM-1 also reduced the efficiency of transfer infection by 60 to 70%, whilst antibodies to αVβ6 integrin had no significant affect on the efficiency of transfer infection (Figure 3A). These results suggest that the interaction between B cells and epithelial cells under these experimental conditions is largely mediated by the interaction of LFA-1 on B cells with ICAM-1 on epithelial cells.

Certain integrins, particularly α5, αV and α8 recognise an RGD motif within their ligands and peptides containing this motif can effectively block integrin-ligand interactions. We set up transfer infection where both the virus loaded B cells and the epithelial cells were pre-incubated for 1 hour in binding buffer with a range of concentrations of an RGD peptide or a scrambled peptide. As shown in Figure 3B, transfer infection was not blocked by scrambled peptide, but it was blocked by up to 42% with the RGD peptide in a dose-dependant manner. This suggests that an RGD-using integrin, in addition to LFA-1 may mediate the firm association between the B cells and epithelial cells. An alternative explanation would be that EBV itself encodes an RGD-expressing ligand for an epithelial cell integrin that is necessary for infection; this possibility was investigated in additional experiments described later in this report.

B cells also express molecules that interact with components of the epithelial cell basement membrane, including fibronectin and sulphated glycosaminoglycans (GAGs) such as heparan sulphate. For example, CD48, which co-caps with EBV following B cell infection and is present at the viral synapse (Table 1, and data not shown), has been shown to interact with heparan sulphate [23]. In the absence of an available blocking antibody to CD48, we examined whether incubation of the cells with heparan sulphate could inhibit B cell interaction with the epithelial cells, and thereby transfer infection. Figure 3C shows that heparan sulphate inhibits transfer infection by up to 52% in a dose-dependant manner, whilst equivalent concentrations of another sulphated GAG (chondroitin sulphate) and a non-sulphated GAG, (hyaluronic acid) showed no inhibition.

### Transfer infection of polarized epithelial cells is restricted to the basolateral surface

Whilst the preceding experiments provided important clues to the potential molecular mechanisms of B cell/epithelial cell conjugate formation during transfer infection, they do not take into consideration that epithelial cells *in vivo* are polarized. Polarization involves the segregation of different sets of molecules to either the apical or the basolateral surface of epithelial membranes, raising the possibility that the ability of B cells to form conjugates and mediate transfer infection will be influenced by the distribution of adhesion molecules and other receptors at each epithelial surface.

**Figure 4:**
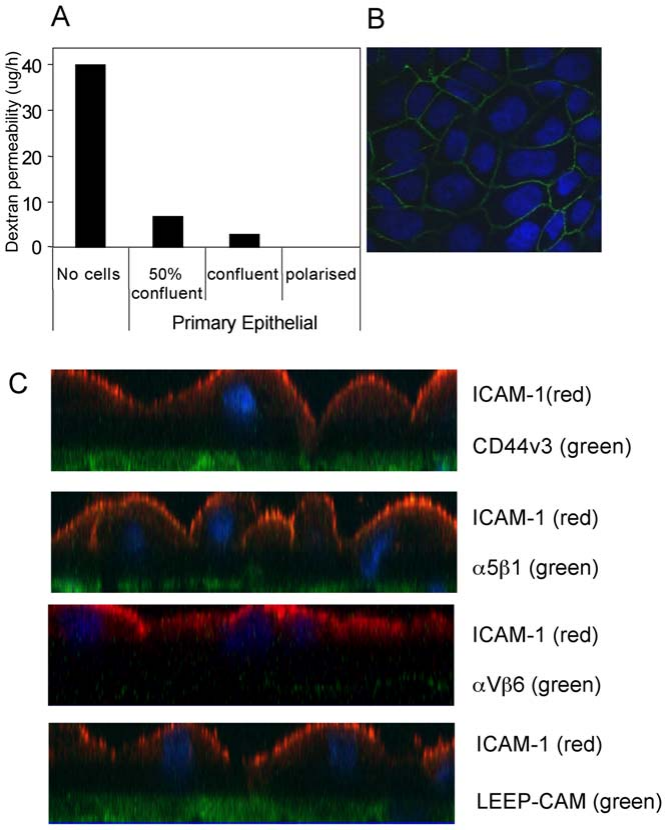
Polarization of primary tonsillar epithelial cells. (A) Primary tonsillar epithelial cells were seeded in triplicate onto 8.0 µM pore-size Transwells. FITC-dextran was added to the medium in the upper reservoir at 50% confluence, fully confluent and 6 days post confluence (polarized). After 3 hours, aliquots of the medium from the lower (basolateral) chamber was analysed for the concentration of FITC-Dextran. The results are plotted as the mean of triplicate samples for each condition. (B) Polarized primary tonsillar epithelial cells were fixed in ice-cold methanol and stained with anti-E-cadherin antibody and visualised with anti-mouse AlexaFluor 488 secondary antibody. Confocal analysis through 1 µm X-Y sections show the staining is localised to the upper lateral membrane. (C) Polarized primary tonsillar epithelial cells were fixed in ice-cold methanol and stained with mAbs to ICAM-1, CD44v3, α5β1 and αVβ6 and LEEP-CAM at both surfaces to determine which epithelial surface these molecules are expressed upon. The cells were examined by confocal microscopy and are represented as X-Z sections.

We therefore set up cultures of polarized primary epithelial cells using previously established methods [24]. The epithelial cells were grown on transwell membranes with 8.0 µm pores, allowing B cell access to both the apical and basolateral surfaces of the epithelial cells. Cell polarization was monitored by measuring the permeability of the cultures to diffusion of a 4 kDa FITC-labelled dextran and the localization of Zo-1 and E-cadherin. FITC-dextran was applied to the upper chamber of the transwell and the concentration in the lower chamber was measured over time. The dextran flux rate in empty transwells was 40 µg/hr. At 50% and 100% confluence, the flux rate was reduced to 5.8 µg/hr and 2.2 µg/hr respectively. Following a further 6-8 days culture of the confluent monolayers, the dextran flux was completely blocked (Figure 4A). At this time, the expression of E-cadherin, essential for the formation of adherens junctions and Zo-1, essential for adherens and tight junctions, were totally localized to the upper lateral cell membranes (Figure 4B), confirming the presence of a polarized monolayer after 10 days of culture.

We next monitored the cellular location of a range of adhesion molecules on polarized epithelial cells, including those suspected from earlier experiments to be involved in B cell/epithelial cell interaction during transfer infection. Cultures of polarized epithelial cells were fixed in ice cold methanol, and both the apical and basolateral surfaces were stained with mAbs to ICAM-1, CD44v3, αVβ6, α5β1, Laminin β1 (LAMB-1) and lymphocyte-endothelial-epithelial cell adhesion molecule (LEEP-CAM). The epithelial cells were examined by confocal microscopy (Figure 4C). Interestingly, expression of ICAM-1, which was shown to be involved in transfer infection of conventional monolayer epithelial cultures (Figure 3), was restricted to the apical surface of polarized cells. In contrast to ICAM-1, the heparan sulphated CD44v3, αVβ6 and α5β1 integrins, LAMB-1 and LEEP-CAM were all specifically expressed on the basolateral surface.

**Figure 5:**
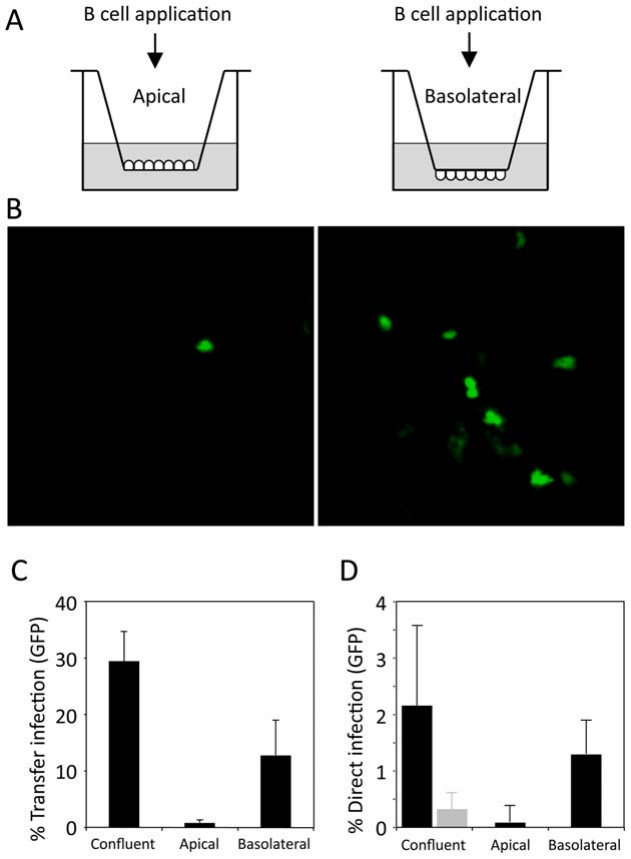
Transfer infection of polarised epithelial cells. (A) Primary tonsillar epithelial cells were grown until polarized on 8 µm pore-size Transwell inserts, as illustrated, to allow access to the apical or basolateral surface of the epithelial cells by B cells. (B) EBV-infected B cells were added to either surface for 4 hours, then washed off and epithelial cell infection observed after 24 hours by GFP-expression. (C) Epithelial cell infection (GFP) was quantitated by flow cytometry of >10,000 cells. The % transfer infection (mean of triplicates) was plotted for confluent (unpolarized) cells and compared to transfer infection from the apical and basolateral surfaces. (D) Direct epithelial cell infection with the gp350-deleted virus. Infection (GFP) was quantitated by flow cytometry of >10,000 cells. The % of direct infection (mean of triplicates) for confluent (unpolarized) cells was compared to direct infection from the apical and basolateral surfaces. The gp350-restored virus is also shown (grey bar).

By seeding primary tonsillar epithelial cells on either the upper or lower surface of the transwell membrane, EBV-loaded B cells or cell-free virus could be applied to either the apical or the basolateral surface of polarized epithelial cell monolayers (see schematic in Figure 5A). As with conventional epithelial cell cultures, infection with cell-free virus at either surface of polarized cells resulted in very poor (<0.1%) efficiency of infection (data not shown). Similarly, addition of virus-loaded B cells to the apical membrane resulted in less than 0.5% infection (Figure 5B, left panel, C). Furthermore, when these infected monolayers were examined by confocal microscopy, the GFP-positive cells were in fact non-polarized cells growing above the polarized monolayer (data not shown). In contrast, exposure of virus-loaded B cells to the epithelial cell basolateral surface increased the efficiency of infection (Figure 5B, right panel); confocal microscopy confirmed that every GFP-positive cell was within the polarized monolayer, and flow cytometry of the isolated epithelial cells typically showed up to 20% of cells expressing GFP (Figure 5C). Infection of confluent or polarized epithelial cells by direct infection using the virus deleted for gp350 showed the same pattern of infection as that of transfer infection, with the virus entering the epithelial cells predominantly via the basolateral surface (Figure 5D).

As ICAM-1 is located at the apical surface of polarized epithelial cells, these results strongly suggest LFA-1 and ICAM-1 play no role in transfer infection of polarized epithelial cells *in vivo*. Furthermore, the results in Figures 5C and 5D also suggest that the EBV receptor for epithelial cells is differentially expressed on the basolateral surface.

### B cell adhesion to the basolateral membrane is mediated by heparan sulphate, CD11b and LEEP-CAM

**Figure 6:**
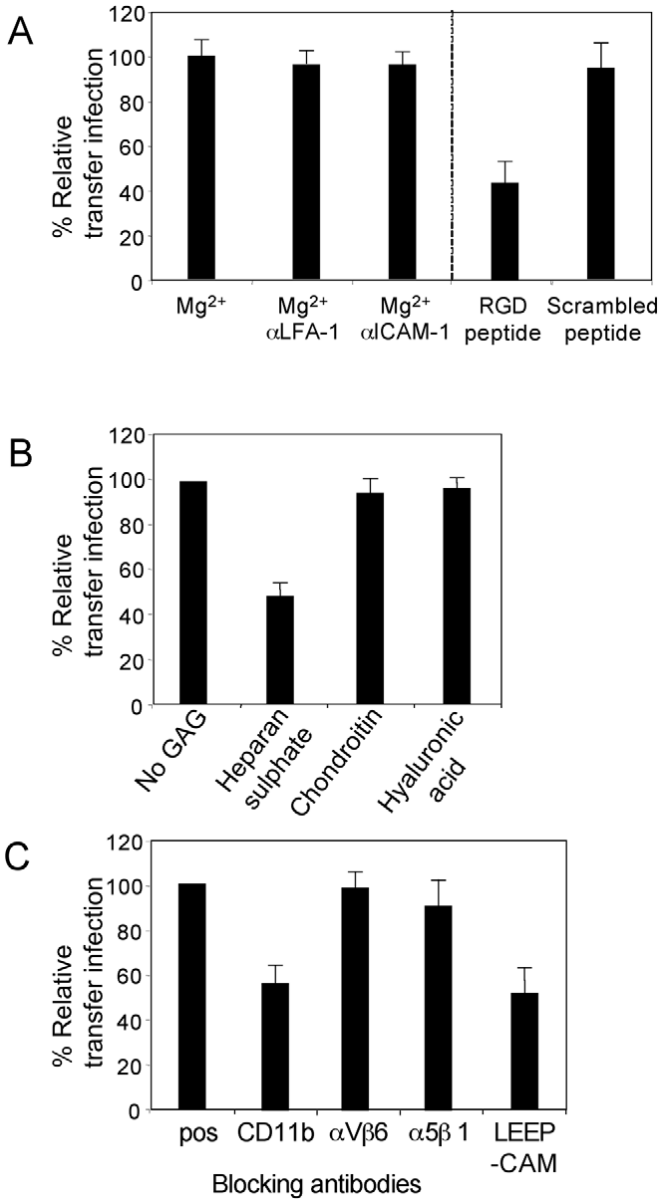
B cell adhesion to the epithelial cell basolateral surface. Primary tonsillar epithelial cells were plated onto 8 µM pore-size Transwells and grown until confluent. The polarized epithelial cells were examined by transfer infection via the basolateral surface for cation, RGD or GAG usage. (A) EBV-infected B cells (24 h p.i.) and epithelial cells were washed in Ca^2+^/Mg^2+^-free PBS. As above, co-culture was performed in binding buffer in the absence of cations (EDTA) or the presence of Mg^2+^, then under optimal cation conditions, B cells and epithelial cells were pre-incubated in blocking antibodies to LFA-1, ICAM-1, RGD peptide or scrambled peptide. Co-culture was performed for 1 hour, then B cells washed off. The epithelial cells were trypsinised off the transwell membrane and plated onto 24 well plates. Infection was monitored by flow cytometry of >10,000 cells for GFP after 24 hours and plotted as % infection (mean of triplicates) relative to the optimal cation conditions. (B) Under optimal conditions, the EBV-infected B cells (24 h p.i.) were pre-incubated with the GAGs: heparan sulphate, chondroitin sulphate and hyaluronic acid. Transfer infection was performed and analysed as in 6A and plotted as % infection (mean of triplicates) relative to the no GAG control. (C) EBV-infected B cells (24 h p.i.) were pre-incubated with (10–20 µg/ml) blocking antibodies to CD11b, αVβ6 and β1 integrins and LEEP-CAM before co-culture for 1 hour with polarized epithelial cells. The B cells were washed off and epithelial cells re-plated, as above. Epithelial infection was monitored by GFP expression after 24 hours by flow cytometry of >10,000 cells and plotted as % infection (mean of triplicates) relative to the positive control.

Given that ICAM-1 was differentially expressed on the apical surface of polarized epithelial cells, whilst transfer infection occurred at the basolateral surface, we re-examined the role of integrins, or other components of the extracellular matrix, in transfer infection. First, we examined the contribution of cations and RGD to the B cell interaction under polarized epithelial conditions. Both virus-loaded B cells and polarized epithelial cell monolayers were washed twice in Ca^2+^/Mg^2+^-free PBS, then co-cultured in the presence of EDTA (no cations) or with EGTA and Mg^2+^ ions restored. In addition, replicate cultures were pre-incubated with blocking mAbs to LFA-1 and ICAM-1 in EGTA plus Mg^2+^ as above, before application to the epithelial basolateral surface. GFP expression was analysed by flow cytometry after 24 hours. In contrast to transfer infection of non-polarized cells (Figure 3A), infection via the basolateral surface was unaffected by the presence of blocking antibodies to LFA-1 or ICAM-1 (Figure 6A). Consistent with the effects of cation depletion, the efficiency of transfer infection was significantly blocked by RGD peptide but unaffected by a scrambled control peptide (Figure 6A). This suggests that transfer infection via the basolateral surface is mediated by RGD-requiring integrins. However, it should be noted that these assays cannot distinguish between the cell-cell interaction and the binding/internalization of EBV to the epithelial cell.

CD44v3, an epithelial-specific cell surface molecule whose expression is restricted to the basolateral surface of polarized cells (Figure 4C), contains side-chains modified by heparan sulphate moieties [25]. We therefore asked whether heparan sulphate, as it did with unpolarized monolayer cultures (Figure 3B), played a role in transfer infection via the basolateral surface. Virus-loaded B cells were preincubated with 32 µg/ml of heparan sulphate, chondroitin sulphate or hyaluronic acid in binding buffer for 1 hour at 37°C before co-culture with the epithelial basolateral surface. The epithelial cells were analysed for GFP expression after 24 hours and, as shown in Figure 6B, heparan sulphate inhibited transfer infection by up to 55% whilst the control GAGs, chondroitin and hyaluronic acid, had no effect.

Integrins and heparan sulphate side-chains appear to play a significant role in transfer infection via the epithelial basolateral membrane. We therefore examined the effects of blocking antibodies to integrins expressed on epithelial cells and to receptors on B cells that bind heparan sulphate. In each experiment, the virus-loaded B cells and the epithelial cells were pre-incubated with the antibodies for 30 minutes at 4°C, co-cultured in binding buffer containing the antibodies, B cells removed as above and infection monitored by flow cytometric analysis of GFP. As shown in Figure 6C, the mAbs to the heparan sulphate-interacting CD11b expressed on B cells, and the LEEP-CAM integrin expressed on epithelial cells both independently caused a significant reduction in transfer infection by up to 45%; in contrast, blocking mAbs to αVβ6 and α5β1 integrins did not inhibit transfer infection.

### EBV infection via the basolateral surface requires gp110 and gH

**Figure 7:**
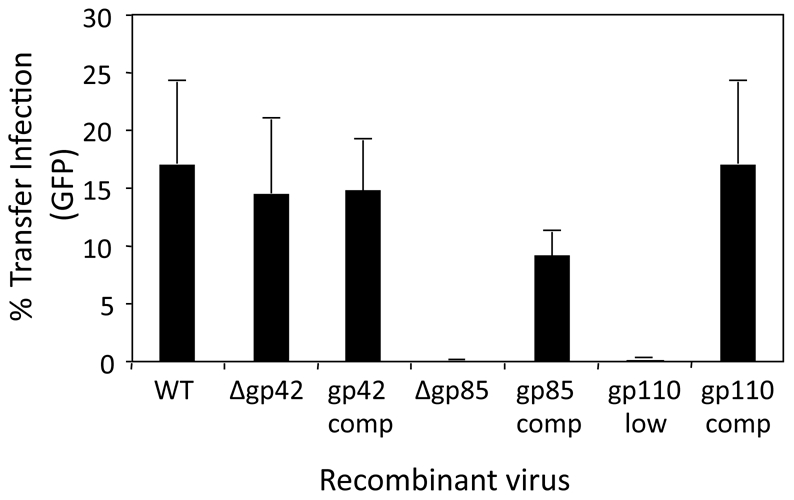
Virus requirements for basolateral surface entry. Transfer infection was performed with B cells infected for 24 h with recombinant EBV carrying deletions (Δ) for the glycoproteins gp42 and gp85, plus the WT virus without reconstituted gp110 (gp110 low), and their respective complemented (restored) glycoprotein counterparts. The B cells were co-cultured with polarized primary tonsillar epithelial cells at the basolateral surface for 1 hour, washed off and the epithelial cells trypsinised from the transwell and re-plated. Epithelial infection was monitored by flow cytometric analysis of GFP expression after 24 hours and plotted as % infection. Due to the inherent variability in infection between donor epithelial cells, assays were performed on triplicates of the same donor cells.

The experiments described so far aimed to identify cellular components of the interaction between virus-loaded B cells and epithelial cells. These experiments also provided evidence for virus-specific interactions with cellular components, which may contribute to adhesions and/or mediate essential fusion events required for virus entry from the synapse into the epithelial cell. Thus, while LFA-1/ICAM-1 adhesions are sufficient to facilitate B cell/epithelial cell conjugates and transfer infection of non-polarized epithelial cells, they were not able to facilitate transfer infection via the apical surface of polarized epithelial cells; suggesting that a critical cellular receptor for EBV is polarized to the basolateral surface. To elucidate this further, we first examined a panel of recombinant EBV viruses, each deleted or mutated to knock-out selected envelope glycoproteins, for their ability to mediate infection of epithelial cells via transfer infection to the basolateral surface. As previously reported for transfer infection of non-polarized epithelial cells [11], gp85 (gH) and gp110 were both essential for basolateral infection of polarized cells (Figure 7). Expression of gp42, which binds to HLA class II and is essential for virus entry into B cells [4] was dispensable for transfer infection of HLA-class II-negative epithelial cells (Figure 7).

### Transfer infection via the basolateral surface requires an EBV interaction with integrins and fibronectin

To identify the cellular ligands for EBV on epithelial cells, we needed to simplify the infection model to perform blocking experiments that avoided the complication of intercellular adhesions. We have previously shown that recombinant EBV deleted for the gene encoding the major glycoprotein (gp350) that binds to CD21 on B cells, can directly infect epithelial cells [11]; although less efficient that transfer infection with wild-type EBV, direct infection with cell-free Δgp350 recombinant virus is substantially more efficient than direct infection with wild-type EBV. Importantly, Δgp350 recombinant virus infection of polarized epithelial cells was observed only via the basolateral surface (Figure 5D), suggesting that EBV/epithelial cell interactions with cell-free Δgp350 recombinant virus are likely to reflect those operating during transfer infection with wild-type EBV.

**Figure 8:**
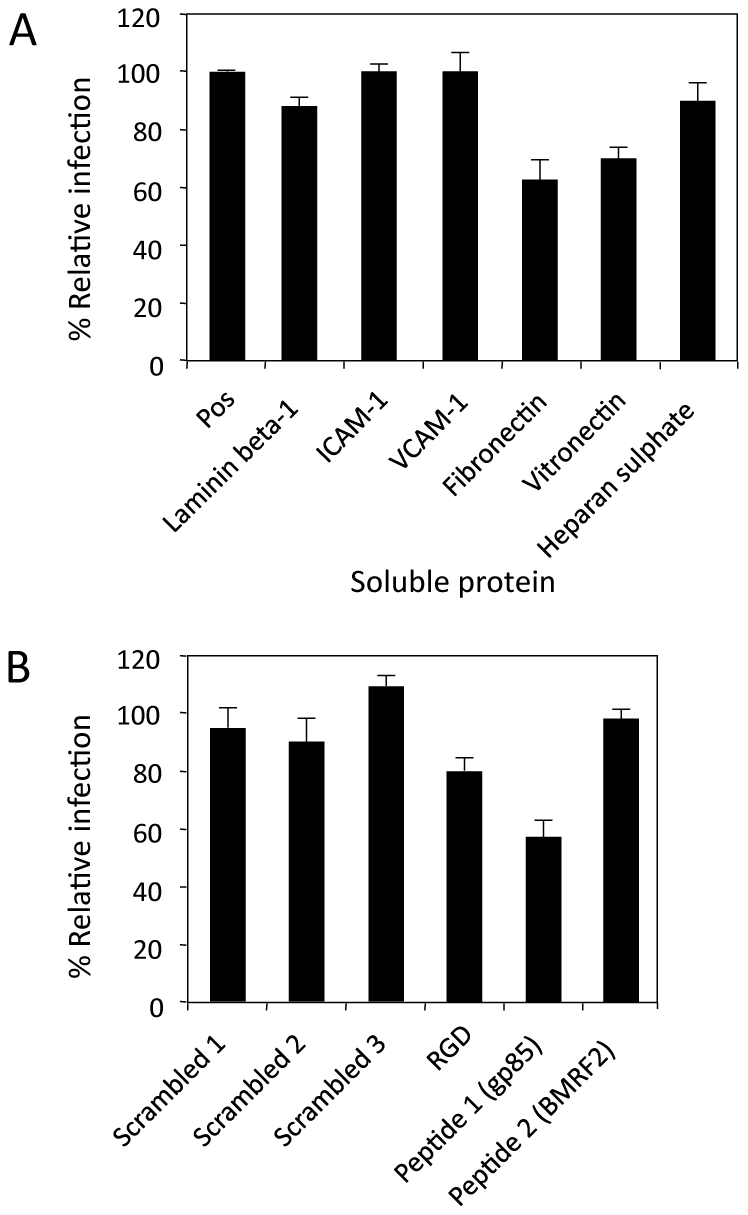
Inhibition of direct polarised epithelial cell infection. Recombinant EBV carrying a deletion for gp350 (Δgp350) was used to directly infect polarized tonsillar epithelial cells from the basolateral surface. (A) Purified Δgp350 at an MOI 100 was pre-incubated for 1 hour at 37°C with proteins of the extracellular matrix: laminin β-1, fibronectin, vitronectin, heparan sulphate, or expressed on the surface of epithelial cells: ICAM-1 or VCAM-1 as a control. The virus was then incubated for 1 hour with the epithelial cells. Epithelial infection was monitored by flow cytometry for GFP and plotted as % infection (mean of triplicates) relative to the positive control. (B) The Δgp350 virus was pre-incubated with an RGD peptide, a peptide containing the KGD motif specific to gp85 and a peptide containing an RGD motif specific to BMRF2, plus control scrambled peptides in binding buffer for 1 hour at 37°C. The virus was then incubated for 1 hour with the epithelial cells. Epithelial infection was again monitored by flow cytometry for GFP expression and plotted as % infection relative to a positive control without peptides.

A yeast-2-hybrid screen reported by another group [26] suggested that gp110 interacted with laminin beta-1 (LAMB1) and fibronectin. We therefore pre-incubated purified Δgp350 recombinant virus with LAMB1, fibronectin, vitronectin, heparan sulphate, ICAM-1 or VCAM-1 for 1 hour at 37°C before adding to the basolateral surface of polarized epithelial cells. Infection was monitored by flow cytometric analysis of GFP expression after 48 hours. Fibronectin and vitronectin reproducibly inhibited infection in these experiments by up to 40%, whilst LAMB1, ICAM-1, VCAM-1 or heparan sulphate did not affect the efficiency of infection (Figure 8A). The lack of effect of heparan sulphate on direct virus infection strengthens the interpretation that the heparan sulphate inhibition of transfer infection (Figures, 3C and 6B) reflects interference with intercellular CD11b/CD44v3 adhesions. The inhibition of direct virus infection by fibronectin and vitronectin is consistent with them being ligands for EBV glycoproteins, although whether these interactions play a role in the infection process itself is unclear since fibronectin and vitronectin are also components of the extracellular matrix *in vivo* and might therefore act as an inhibitory barrier to infection.

We next re-visited the effects of interfering with possible RGD integrin interactions during direct infection with Δgp350 recombinant virus, since the inhibition of transfer infection using inhibitors of integrins (Figures. 3B and 6A) may inhibit EBV/epithelial cell interactions as well as B cell/epithelial cell interactions. We pre-incubated purified Δgp350 with a consensus control RGD peptide, an RGD 13-mer peptide specific to BMRF2 (IFCARGDHSVASL), a KGD 13-mer peptide specific to gH/gp85 (RVTEKGDEHVLSL), or with scrambled peptides. The virus was then applied to the basolateral surface of the polarized epithelial cells and infection monitored as above. The RGD peptide showed a small (20%) but reproducible inhibition of infection, whilst the BMRF2 specific RGD peptide showed no inhibition (Figure 8B). In contrast, the gH/gp85-specific KGD peptide reproducibly inhibited infection by up to 40%. This suggests that gH, interacts with an integrin to mediate viral entry via the basolateral surface.

### Transfer infection via the basolateral surface of epithelial cells is predominantly mediated by memory B cells

**Figure 9:**
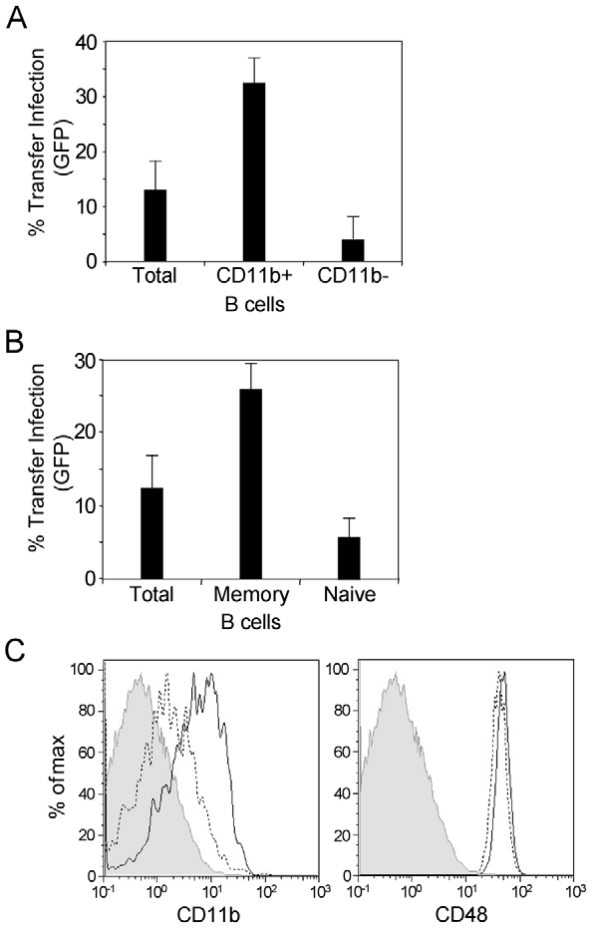
Transfer infection via the basolateral surface is mediated via memory B cells. (A) Total B cells, CD11b +ve and CD11b -ve B cells were sorted and infected with EBV (24 h p.i.) and co-cultured at the basolateral surface of polarized primary tonsillar epithelial cells for 1 hour. The B cells were washed off and the epithelial cells monitored for infection by flow cytometric analysis of GFP expression after 24 hours. Infection was plotted as the mean of triplicates. (B) Total B cells, memory B cells and naïve B cells were sorted, infected with EBV, and co-cultured with the polarized epithelial cells as above. Infection was monitored by flow cytometric analysis of GFP expression and plotted as the mean of triplicates. (C) Flow cytometric analysis of naïve and memory B cell expression of CD11b and CD48. Total B cells were stained with CD27 and IgD plus CD11b or CD48. The shaded area represents the isotype control; the dotted line represents the naïve B cells (IgD^+^CD27^−^); the solid line represents the memory B cells (IgD^−^CD27^+^).

Given that blocking mAb to CD11b significantly reduced the efficiency of transfer infection, suggesting this molecule may play a role in the interaction between the virus-loaded B cell and the epithelial basolateral surface, we asked whether the CD11b^+^ subset of B cells would show enhanced efficiency of transfer infection. We initially stained resting B cells with a non-blocking CD11b mAb and isolated CD11b^+^ and CD11b^−^ cells by fluorescence-activated cell sorting. The sorted cells were incubated with virus for 18 hours and transfer infection performed via the basolateral surface. The results in Figure 9A show that CD11b^+^ B cells were more efficient than unsorted B cells at mediating transfer infection and 4 to 5-fold more efficient than were CD11b^−^ cells.

Since CD11b^+^ cells originate from the memory B cell subset, memory and naïve B cells populations were sorted according to their CD27 and IgD expression. As shown in Figure 9B, memory B cells (CD27^+^IgD^−^) showed enhanced transfer infection compared to total B cells, and were 4- to 5-fold more efficient than naïve B cells (CD27^−^IgD^+^). As both CD48 and CD11b have been reported to interact with heparan sulphate, we examined CD48 expression in control and virus-loaded memory B and naïve B cells at the time (24 hours post-infection) that they were used for transfer infection. The expression of CD48 did not differ between the memory and naïve B cell subsets (Figure 9C), suggesting that although CD48 has the potential to interact with heparan sulphate, and hence with CD44v3 of epithelial cells, it is not responsible for the enhanced transfer infection imparted by the memory subset.

## Discussion

In this study, we have shown that binding of EBV to CD21 on B cells causes activation of adhesion molecules that enable firm heterotypic adhesions to be formed with epithelial cells, providing conditions for efficient virus transfer to infect epithelial cells. Our initial attempts to identify the cellular and viral components involved in synapse formation and transfer infection suggested an apparently critical role for B cell LFA-1/epithelial ICAM-1 interactions, at least in conventional monolayer cultures of primary epithelial cells. Since expression of ICAM-1 is restricted to the apical surface of polarized epithelial cells *in vivo*, these results questioned the physiologic significance of transfer infection. However, through the use of an *in vitro* model of polarized primary epithelial cells, we showed that transfer infection could be demonstrated only *via* the basolateral surface, and did not involve LFA-1/ICAM-1 interactions. Instead, B cell CD11b/epithelial cell CD44v3 interactions are an important component of intercellular adhesion, and interaction of viral gH/gp85 with epithelial cell integrins, including but not exclusively αVβ6, is required for viral entry into epithelial cells during transfer infection.

CD21 is both the receptor for C3d and for the EBV major glycoprotein gp350. Each ligand binds CD21 through distinct sites but both trigger B cell activation and proliferation, independently of CD19 or BCR co-ligation [27], [28]. Activation of B cells *in vivo* induces their recirculation and homing of to effector sites; intrinsic to this process is the activation of B cell surface adhesion molecules required for extravasation and migration. Within the effector site, B cell activation is likely to enable firm adhesion of B cells to epithelial cells. Although B cell interactions with epithelial cells are not as well studied as other lymphocyte/epithelial cell interactions, they are observed *in vivo*. Thus, B cells are commonly found underlying the epithelium of tonsillar crypts and often infiltrate the whole epithelial cell thickness [29], [30]. In addition, IgA-secreting plasma cells are commonly found in the lamina propria underlying the mucosal epithelium within the mucosa-associated lymphoid tissue (MALT) [29], [30]. MALT is defined as the organised lymphoid tissue in the nasal or gut mucosa with aggregates of lymphoid-like follicles and lymphocyte infiltration of the overlying epithelium [31].

Our *in vitro* experiments showed that firm adhesion of B cells with epithelial cells required the activation of B cell adhesion molecules in a tetraspanin-mediated process. The major role of tetraspanins is to organise other proteins into signal transducing complexes at the cell surface, exemplified by interactions between tetraspanins and the widely-expressed β1 integrins [32]. The β1 integrins, through their molecular interactions with extracellular-matrix, particularly fibronectin, and cell-surface proteins, have a crucial role in leukocyte adhesion, migration, differentiation and proliferation [33]. Following ligation of CD21 by EBV on primary resting B cells, we observed co-capping of β1 integrins, together with CD11b, CD47, CD49d, LFA-1, and αVβ6 integrins, with the tetraspanins, CD63, CD81 and CD82. Capping of integrins causes an increase in avidity of the integrin for its ligand [34], [35], [36]. Full activation of integrins also requires requires a stoichiometric alteration to the extended form of the molecule [37], [38]. That integrin activation occurs following ligation of CD21 with EBV or antibody, was demonstrated (data not shown) through the use of an mAb recognising the activated conformation of LFA-1 [39] and through the ability of such cells to form LFA-1/ICAM-1 mediated firm adhesions with unpolarized monolayer epithelial cells (Figure 3). Although LFA-1 itself is not involved in transfer infection via the basolateral surface of polarized epithelial cells, its activation is indicative of EBV-induced activation of B cell integrins.

A role for activation of β1 integrins is implicated by an inhibition of both conjugate formation and transfer infection observed following treatment of the B cells with fibronectin immediately prior to co-culture with epithelial cells (data not shown). The interaction of lymphocytes with the extracellular matrix beneath the basolateral surface of epithelium, particularly with fibronectin, has been extensively studied, as indeed has the interaction of lymphocytes with endothelial cells in high endothelial vessels for transendothelial migration. However, very little information is available on the interaction of B cells with epithelial cells themselves. In addition to a possible role for β1 integrins, we showed in the present study that the B cell/epithelial cell interaction involves heparan sulphate moieties of molecules polarized at the basolateral membrane (Figure 6). Many epithelial cell basolateral molecules have heparan sulphate modifications including syndecan-1 and CD44v3. Our study was not designed to exhaustively test every heparan sulphated molecule. However, the heparan sulphate proteoglycan form of CD44v3 is of particular interest as it is epithelial cell specific. Two molecules, CD48 and CD11b, expressed on human B-lymphocytes have previously been shown to interact with heparan sulphate moieties, and our data implicated a role for the latter in B cell/epithelial cell interactions and transfer infection of polarized epithelial cells. Ideally, the mechanism of B cell-epithelial cell interaction would be confirmed by knockdown experiments on the individual B cell adhesion molecules. However knockdown experiments on primary resting B cells are technically very difficult and inefficient.

Transfer infection of polarized epithelial cells is restricted to the basolateral surface, even though conjugate formation via LFA-1/ICAM-1 interactions is possible at the apical surface (Figure 4C). Furthermore, whilst substantially less efficient than transfer infection, the direct infection of the polarized epithelial cells with cell-free Δgp350 recombinant virus was more efficient *via* the basolateral surface (Figure 5D). Together, these observations strongly suggest that ligands on the epithelial cells that bind to EBV and facilitate fusion and entry of the virus into the epithelial cell are similarly restricted to the basolateral surface. The expression of αVβ6 integrin, previously suggested to be an EBV gH/gp85 fusion ligand [40] is restricted to the basolateral epithelial surface, and blocking assays using a KGD peptide specific to the viral glycoprotein, gH, significantly blocked virus entry into the epithelial cells. The αVβ6 integrin is unlikely to be the sole mediator of fusion since not all infected primary epithelial cells express αVβ6, and some epithelial cell lines such as AdAH, are highly infectable by transfer infection but do not express αVβ6 integrin ([11]; data not shown).

Fibronectin was previously shown to bind to gB (gp110) in yeast 2-hybrid assays [26] and binding of gH/gL complexes to epithelial cells can be inhibited by fibronectin or vitronectin [40]. Fibronectin is therefore another candidate epithelial cell ligand for EBV since gB, gH and gL are all essential EBV glycoproteins required for transfer infection (Figure 7; [11]). However, the physiological significance of that observation is complex. Notwithstanding the fact that direct infection of primary epithelial cells is substantially less efficient than transfer infection [11], cell-free virus *in vivo* would be required to traverse the fibronectin-rich extracellular matrix before it can access the fibronectin on the epithelial cell surface. The extracellular matrix might therefore represent a barrier to epithelial cell infection by cell-free virus. In contrast, interaction of lymphocyte integrins with fibronectin of the extracellular matrix is known to facilitate migration of lymphocytes to the epithelium. Virus bound to B cells would therefore access the basolateral surface of epithelial cells more efficiently than would cell-free virus.

The final observation in our present study is that transfer infection is predominantly mediated by the CD11b+ memory B cell subset. Physiologically, interactions between memory B cells and epithelial cells occur at mucosal effector sites, namely sites where antibodies are released in response to continual exposure to viruses and bacteria. Indeed, the reticular epithelium of tonsillar crypts form pockets filled with Pax5-expressing B cells [41]. The recruitment of plasma cells and memory B cells is a well-established contribution of epithelial cells to mucosal immunity; furthermore, epithelial cells can also recruit pre-switched B cells to the subepithelial region and induce them to switch and differentiate to produce antibody [41], [42]. This provides an environment where latently-infected B cells recruited to the subepithelial region may be induced to lytic virus replication concomitant with plasma cell differentiation. It is possible that virus-producing plasma cells might themselves form firm adhesions with the basolateral surface of the epithelial cells. However, in an environment where a rare virus-producing cell is surrounded by memory B cells, it is more likely that epithelial cell infection occurs predominantly through memory B cells mopping up released virus and transferring it to epithelial cells by the mechanisms described in this paper.

The physiological role of transfer infection is most likely to be in the production of virus secreted into the oropharynx during established persistent infection of the host. The molecular mechanisms characterised in the present study provide no obvious role for either transfer infection or direct cell-free virus infection of epithelial cells during primary infection of the host.

Our results also impact on how EBV might infect premalignant epithelial cells and contribute to the pathogenesis of nasopharyngeal carcinoma and some cases of gastric carcinoma. If transfer infection can mediate efficient infection of normal epithelia, it is likely that it will also mediate infection of pre-malignant cells, although the outcome of infection may differ; e.g., expression of transformation-associated viral genes and a block on lytic cycle activation in pre-malignant cells might trigger oncogenesis. However, infection of normal and pre-malignant cells may differ in another respect. Normal epithelia are formed of polarized cells in which different molecules are located to the apical and basolateral surfaces [43], [44]. In contrast, pre-malignant epithelial lesions are mostly non-polarized. Therefore, the distribution of adhesion molecules and potential virus receptors will differ in normal *vs* pre-malignant tissue. Our results with non-polarized monolayer cultures (Figures 1-3) allow the possibility that strong adhesions mediated via ICAM-1/LFA-1 may contribute to transfer infection of non-polarized pre-malignant cells but not of polarized normal epithelia.

In summary, this work reconciles a number of paradoxical observations to provide a mechanistic and physiological explanation for EBV infection of non-malignant epithelial cells. As has been suggested previously, normal persistence of EBV in the healthy infected host relies on its success in manipulating normal B cell physiology for its own ends [45]. It would appear that this principle also extends to EBV taking advantage of normal B cell interactions with epithelial cells that are triggered by activation of the complement receptor.

## Supporting Information

Figure S1 Analysis of GFP expression in primary B cells and epithelial cells. Following co-culture of virus-loaded B cells with epithelial cells, the B cells were washed off and both sets of cells analysed after 24 hours for size differentiation by FS and SS, and for GFP expression. GFP expression was evident in only the epithelial cells at the time of analysis for all subsequent experiments.
(0.43 MB TIF)
