# Supplementary figures and images for: Epstein-Barr Virus Infection of Polarized Epithelial Cells via the Basolateral Surface by Memory B Cell-Mediated Transfer Infection
Source: PLoS Pathog. 2011 May 5;7(5):e1001338. doi: 10.1371/journal.ppat.1001338 (PMC3088705; doi:10.1371/journal.ppat.1001338)

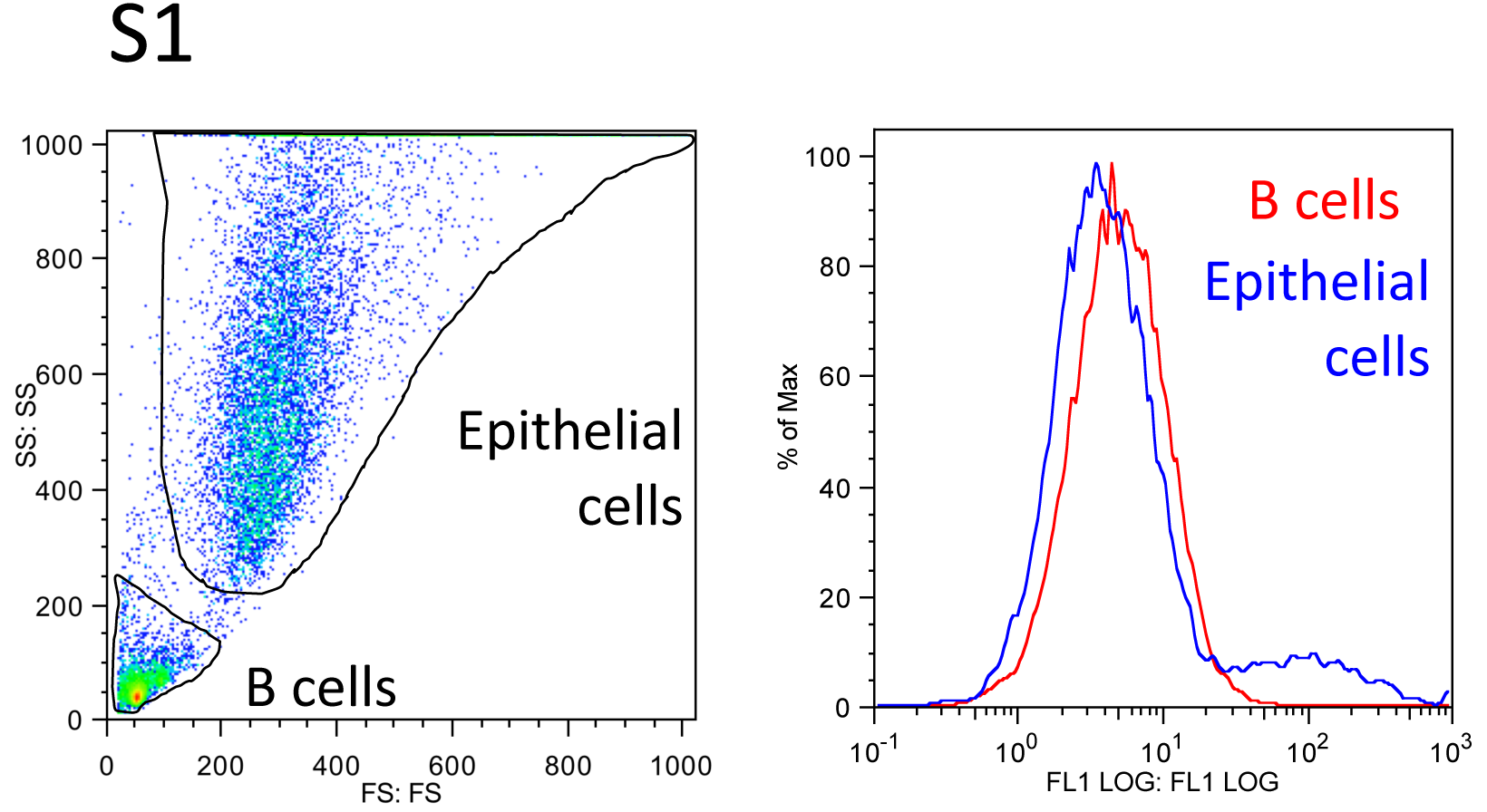

Supplement: Figure S1 — Analysis of GFP expression in primary B cells and epithelial cells. Following co-culture of virus-loaded B cells with epithelial cells, the B cells were washed off and both sets of cells analysed after 24 hours for size differentiation by FS and SS, and for GFP expression. GFP expression was evident in only the epithelial cells at the time of analysis for all subsequent experiments. (0.43 MB TIF) [file ppat.1001338.s001.tif]
